# Role of SLC7A5 in Metabolic Reprogramming of Human Monocyte/Macrophage Immune Responses

**DOI:** 10.3389/fimmu.2018.00053

**Published:** 2018-01-25

**Authors:** Bo Ruem Yoon, Yoon-Jeong Oh, Seong Wook Kang, Eun Bong Lee, Won-Woo Lee

**Affiliations:** ^1^Department of Microbiology and Immunology, Seoul National University College of Medicine, Seoul, South Korea; ^2^Division of Rheumatology, Department of Internal Medicine, Seoul National University College of Medicine, Seoul, South Korea; ^3^Department of Internal Medicine, Chungnam National University School of Medicine, Daejeon, South Korea; ^4^Department of Biomedical Sciences, Seoul National University College of Medicine, Seoul, South Korea; ^5^Cancer Research Institute, Seoul National University College of Medicine, Seoul, South Korea; ^6^Ischemic/Hypoxic Disease Institute, Seoul National University College of Medicine, Seoul, South Korea; ^7^Institute of Infectious Diseases, Seoul National University College of Medicine, Seoul, South Korea; ^8^Seoul National University Hospital Biomedical Research Institute, Seoul, South Korea

**Keywords:** monocyte, macrophage, leucine, mTOR complex 1, glycolysis, rheumatoid arthritis, IL-1β

## Abstract

Amino acids (AAs) are necessary nutrients which act not only as building blocks in protein synthesis but also in crucial anabolic cellular signaling pathways. It has been demonstrated that SLC7A5 is a critical transporter that mediates uptake of several essential amino acids in highly proliferative tumors and activated T cells. However, the dynamics and relevance of SLC7A5 activity in monocytes/macrophages is still poorly understood. We provide evidence that SLC7A5-mediated leucine influx contributes to pro-inflammatory cytokine production *via* mTOR complex 1 (mTORC1)-induced glycolytic reprograming in activated human monocytes/macrophages. Moreover, expression of SLC7A5 is significantly elevated in monocytes derived from patients with rheumatoid arthritis (RA), a chronic inflammatory disease, and was also markedly induced by LPS stimulation of both monocytes and macrophages from healthy individuals. Further, pharmacological blockade or silencing of SLC7A5 led to a significant reduction of IL-1β downstream of leucine-mediated mTORC1 activation. Inhibition of SLC7A5-mediated leucine influx was linked to downregulation of glycolytic metabolism as evidenced by the decreased extracellular acidification rate, suggesting a regulatory role for this molecule in glycolytic reprograming. Furthermore, the expression of SLC7A5 on circulating monocytes from RA patients positively correlated with clinical parameters, suggesting that SLC7A5-mediated AA influx is related to inflammatory conditions.

## Introduction

Monocytes and macrophages are mononuclear phagocytes that have a central role in the initiation, modulation, and resolution of inflammation through phagocytosis, cytokine production, generation of reactive oxygen species, and the activation of the adaptive immunity (1). During inflammatory reactions, circulating monocytes are recruited into the inflamed tissue where they mature into inflammatory macrophages (2, 3). It has been reported that monocytes differentiate into heterogeneous macrophage subsets with distinct characteristics such as “pro-inflammatory” M1 or “pro-resolving” M2 macrophages, depending on the type of stimulation or the course of inflammation. Thus, it is clear that monocytes and macrophages are involved in the pathogenesis of many acute and chronic inflammatory diseases, including autoimmune disorders (4–8).

Immune responses are energy-demanding biosynthetic processes, and activated monocytes and macrophages must strictly regulate their metabolic programs to meet the demands of participating in an immune response (9, 10). Enhanced glycolysis is considered a hallmark metabolic change in LPS-activated monocytes/macrophages. In macrophages, sufficient ATP and biosynthetic intermediates required to maintain effector functions are efficiently generated by glycolysis-mediated glucose uptake (11–13). In addition to glycolysis, fatty acid synthesis is driven by inflammatory signals leading to cell proliferation and inflammatory cytokine production in M1 macrophages. On the contrary, fatty acid oxidation is triggered by tolerogenic stimuli and is linked to the inhibition of inflammation in M2 macrophages (14, 15).

Immune cell activation is tightly coupled with protein synthesis, which is necessary for cell proliferation and cytokine production, and accordingly, there should be an uptake of amino acids (AAs) during this process (11, 16). In addition to their classical role as building blocks for protein synthesis, AAs are also diverted into metabolic intermediates through catabolic process and participate in a variety of metabolic processes (17, 18). Recently, it has been demonstrated that AAs, such as leucine and arginine, activate the mTOR complex 1 (mTORC1) *via* direct binding to sensing molecules (19, 20). Thus, given the novel role of mTORC1 for glycolytic reprograming in monocytes and macrophages, much attention has recently been paid to the modulatory role of this AA (10, 21).

Expression of the heterodimeric AA transporters that are associated with the heavy chain, SLC3A2 (CD98), has been linked to proliferation in many studies (22). SLC3A2 functions as a chaperone, not a transporter, and dimerizes with several different light chains, such as SLC7A5 (LAT1: the l-type AA transporter 1), SLC7A8 (LAT2), and SLC7A11 (xCT) (23). SLC7A5 and SLC7A8 are major transporters for large neutral amino acids (LNAAs) including essential amino acids (EAAs) such as leucine. Of note, SLC7A5 is predominantly expressed in various cancer cells, implicating its critical role for proliferation, growth, and survival of cells (24), whereas SLC7A8 is mainly expressed in normal tissue. Moreover, sustained uptake of AAs *via* SLC7A5 leads to enhanced tumor growth by regulating full activation of mTORC1 (24–26). Considering the similarity of cellular metabolism between cancer cells and activated immune cells, SLC7A5-mediated influx of AAs is likely to play a modulatory role in activated immune cells.

Recent studies revealed that the TCR-mediated induction of SLC7A5 is a critical metabolic checkpoint for T cells (27). Pharmacological blockade or loss of SLC7A5 dramatically inhibits cell proliferation, differentiation to effector cells, and cytokine production by T cells (28, 29). These defects were in part attributable to reduced uptake of leucine for mTORC1 signaling (29). However, although mTORC1 is known to be important for controlling and shaping the effector responses of myeloid innate immune cells, little is known about SLC7A5-mediated AA influx and its effects on the mTORC1 pathway in monocytes and macrophages. Here, we investigate a regulatory role of SLC7A5 in metabolic reprogramming of human monocytes/macrophages. Our data show that activation-induced SLC7A5 expression in human monocytes and macrophages mediates leucine influx leading to enhanced mTORC1-mediated glycolysis, thereby causing augmented production of IL-1β. Moreover, increased expression of SLC7A5 by peripheral monocytes from rheumatoid arthritis (RA) patients positively correlates with clinical parameters, such as CRP and ESR, suggesting that the SLC7A5-mediated AA influx is related to inflammatory conditions.

## Materials and Methods

### Cell Preparation

The study protocols were approved by the institutional review board of Seoul National University Hospital and Chungnam National University Hospital. Peripheral blood of RA patients and healthy controls (HCs) was drawn after obtaining written, informed consent. The methods were performed in accordance with the approved guidelines (IRB No. 2012-01-024 for Chungnam National University Hospital and IRB No. 1109-055-378, 1306-002-491, and 1403-049-564 for Seoul National University College of Medicine). The patient characteristics of RA patients enrolled in this study and distribution of medication use for them are summarized in Tables 1 and 2. Peripheral blood mononuclear cells (PBMC) were isolated from blood by density gradient centrifugation (Bicoll separating solution; BIOCHROM Inc., Cambridge, UK). Monocytes were positively separated from PBMC with anti-CD14 microbeads (Miltenyi Biotec Inc., Auburn, CA, USA).

**Table 1:** Baseline characteristics of rheumatoid arthritis patients (*N* = 24).

Patient characteristics	Value
Age (years), means ± SD	56.96 ± 12.67
Sex (female/male), *N* (%)	20/4 (83.3/16.7)
Disease duration, years	8.27 ± 5.11
Rheumatoid factor— no. of positive (%)	22/24 (91.7)
Rheumatoid factor titer (IU/ml), means ± SD	102.33 ± 164.72
Anti-citrullinated protein antibody, no. of positive (%)	13/15 (54.2)
Anti-citrullinated protein antibody titer (U/ml), means ± SD	164.98 ± 519.50
ESR (mm/h), means ± SD	49.58 ± 35.81
CRP (mg/dl), means ± SD	2.55 ± 4.11
DAS28 ESR, means ± SD	4.16 ± 1.60

**Table 2:** Distribution of medication use.

Classification of medications	No. (%)
Prednisolone	18/24 (75.0)
Methorexate	15/24 (62.5)
Hydroxychloroquine	11/24 (45.8)
Sulfasalazine	6/24 (25.0)
Leflunomide	3/24 (12.5)
Calcineurin inhibitor	2/24 (8.3)
NSAIDs	13/24 (54.1)
Biologics	6/24 (25.0)

### Cell Culture

Purified monocytes were cultured in RPMI 1640 medium supplemented with 10% fetal bovine serum, 1% penicillin/streptomycin, and 1% l-glutamine. To obtain monocyte-derived macrophage, purified CD14^+^ monocytes were cultured in the presence of recombinant human M-CSF (50 ng/ml; PeproTech, Rocky Hill, NJ, USA) for 6 days. In some experiments, a custom-made media depleted of five EAAs (leucine, valine, isoleucine, phenylalanine, and methionine) were used (Welgene, Kyungsan, Republic of Korea). All AAs (l-leucine, l-valine, l-isoleucine, l-phenylalanine, and l-methionine) for replenishing experiments were purchased from Sigma-Aldrich (St. Louis, MO, USA).

### Transfection of SLC7A5-Targeted siRNA

Human primary macrophages were transfected with 6.6 nM of pre-made human SLC7A5 siRNA (Cat. No. 1027416) and 6.6 nM of control siRNA (Cat. No. 1027280) (both from QIAGEN, Hilden, Germany) by Lipofectamine^®^ RNAiMAX reagent (Thermo Fisher scientific, Waltham, MA, USA). The transfected cells were incubated for 4 h in serum-free RPMI 1640 media, followed by LPS (100 ng/ml)-stimulation for another 20 h before use.

### Immunoblot Analysis

Monocytes, macrophages, and cell lines were washed with ice-cold PBS and lysed in lysis buffer containing protease and phosphatase inhibitor cocktail (Cell Signaling Technology, Danvers, MA, USA). Cell lysates were run on 8% SDS-PAGE gel and transferred onto a PVDF membrane (Bio-Rad, Hercules, CA, USA), which was then blocked for 1 h with 5% BSA in Tris-buffered saline solution (TBS), containing 0.1% Tween 200. The membrane was then incubated overnight at 4°C with anti-human LAT1 (SLC7A5), anti-BCAT, anti-phosphor-p70S6K, anti-p70S6K (all from Cell Signaling Technology), or anti-human LAT2 (SLC7A8) (Abcam, Cambridge, UK), washed, and incubated for 1 h at RT with the HRP-conjugated secondary Ab (membranes were developed by SuperSignal™ West Femto Maximum Sensitivity Substrate) (Thermo Fisher scientific).

### RT-PCR

Total RNA was extracted from freshly isolated or cultured cells using TRIzol reagents (life technologies, Grand Island, NY, USA), and cDNA was synthesized by GoScript reverse transcription system (Promega, Madison, WI, USA). Real-time quantitative RT-PCR was performed in triplicates on a 7500 PCR system (Applied Biosystems, Grand Island, NY, USA) using following primers: *SLC7A5*: 5′-GAAGGCACCAAACTGGATGT-3′ and 5′-GAAGTAGGCCAGGTTGGTCA-3′; *SLC7A8*: 5′-AACCTTCCCAGAGCCATCTT-3′ and 5′-GTGGACAGGGCAACAGAAAT-3′; *BCAT1*: 5′-AAGATGGGAGGGAATTACGG-3′ and 5′-TGGAGGAGTTGCCAGTTCTT-3′; *TNF*: 5′-AGCCCATGTTGTAGCAAACC-3′ and 5′-TGAGGTACAGGCCCTCTGAT-3′; *IL1B*: 5′-CACGATGCACCTGTACGATCA-3′ and 5′-GTTGCTCCATATCCTGTCCCT-3′; and β*-actin*: 5′-GGACTTCGAGCAAGAGATGG-3′ and 5′-AGCACTGTGTTGGCGTACAG-3′. The levels of gene expression were normalized to the expression of β-actin. The comparative CT method (ΔΔCT) was used for the quantification of gene expression.

### Gene Expression Arrays

Total RNA was purified from peripheral CD14^+^ monocytes from RA patients and HCs using the RNeasy mini kit (Qiagen). Microarray analysis was done with human whole genome expression 44K (Agilent Technologies, SantaClara, CA, USA). Heat map analysis was performed using web-based MORPHEUS software (http://software.broadinstitute.org/morpheus).

### Enzyme-Linked Immunosorbent Assay (ELISA)

The amount of TNF-α and IL-1β in culture supernatant was quantified by commercial ELISA kits (Thermo Fisher Scientific). The measurement of OD (Optical density) was performed using the infinite 200 pro multimode microplate reader (Tecan Group Ltd., Seestrasse, Switzerland).

### ^3^H-Amino Acid Uptake Assay

Cells were incubated in HBSS for 10 min after removing culture media. ^3^H-leucine or -methionine (Perkin Elmer, Waltham, MA, USA) uptake assay was initiated by incubating the cells in HBSS containing 0.5–1 μCi for 15 min. Cells were detached with 1 M NaOH after three times washing by ice-cold HBSS. The radioactivity was measured using beta scintillation counter MicroBeta^®^ (Perkin Elmer).

### Metabolic Analysis

To profile metabolic state of the cells, human primary monocytes or macrophages treated BCH or JPH203 in the presence of 100 ng/ml LPS (Sigma-Aldrich) for 24 h were seeded as a monolayer onto XFe24 cell culture plates (Seahorse Bioscience, MA, USA). The culture media were replaced with XF assay media supplemented with l-glutamine (300 µg/ml) and incubated for 1 h in non-CO_2_ incubator. Glucose (10 mM), oligomycin (2 µM), and 2-DG (50 mM, all from Sigma-Aldrich) were sequentially treated into the cells during real-time measurements of extracellular acidification rate (ECAR) and OCR (Oxygen consumption rate) using XFe24 analyzer (Seahorse Bioscience). Glycolysis parameters were calculated using XF glycolysis stress test report generator program which was provided from manufacturer (Seahorse Bioscience). Glycolysis and glycolysis capacity were calculated by subtracting ECAR after glucose treatment from ECAR before oligomycin and subtracting ECAR after oligomycin from ECAR before 2-DG treatment, respectively.

### Statistics

A paired *t*-test, unpaired *t*-test, or Pearson correlation analysis was done to analyze data using Prism 5 software (GraphPad Software Inc., La Jolla, CA, USA) as indicated in the figure legends. *p*-Values of less than 0.05 were considered statistically significant.

## Results

### Elevated SLC7A5 Expression in Activated Human Monocytes and Macrophages

**Figure 1:**
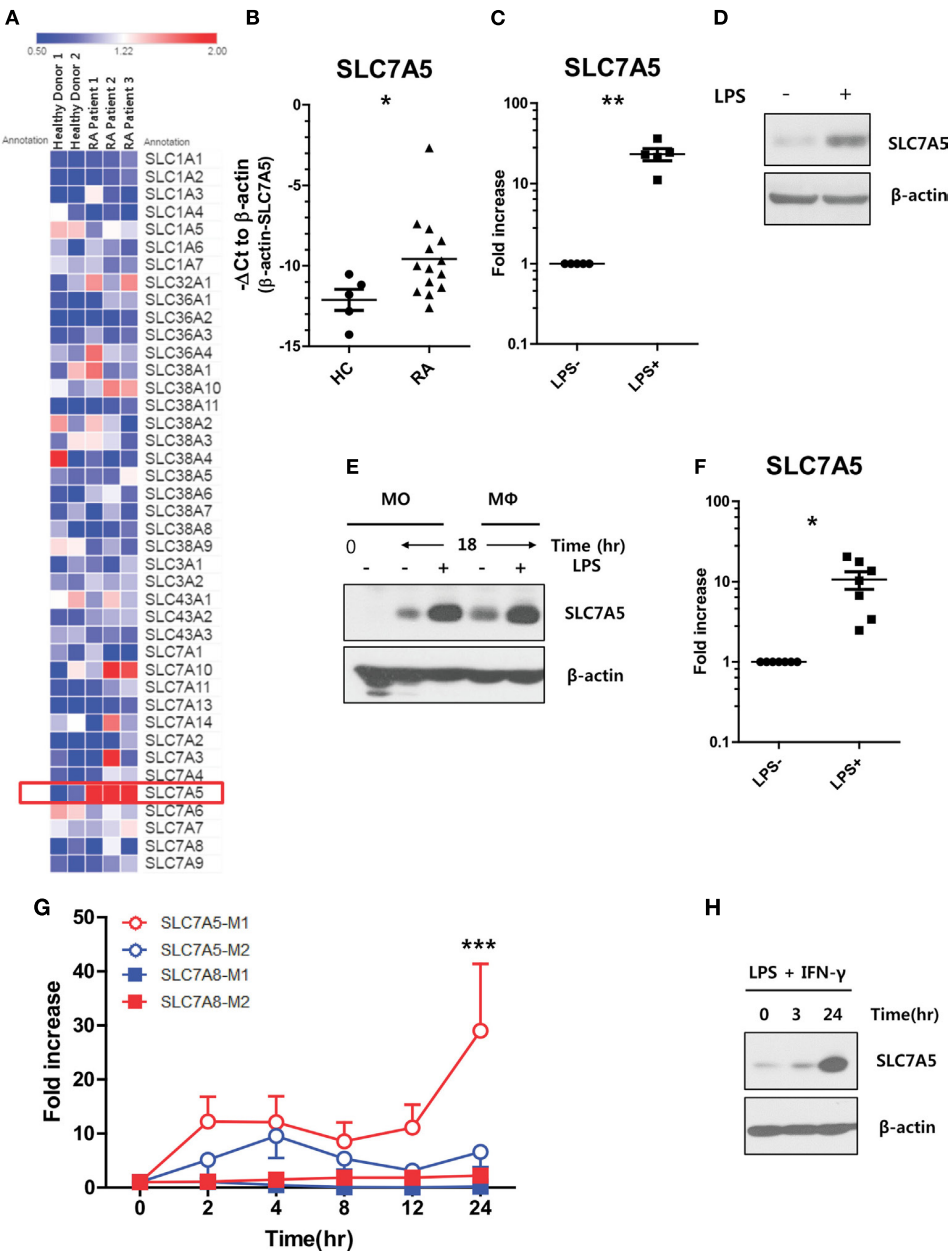
Increased expression of SLC7A5 in activated human monocytes and macrophages. **(A)** Microarray heat map analysis on 41 AA transporters was expressed by peripheral monocytes. CD14^+^ monocytes were purified from peripheral blood mononuclear cells derived from healthy controls (HCs) (*n* = 2) and rheumatoid arthritis (RA) patients (*n* = 3). **(B)** Quantitative PCR analysis of SLC7A5 gene expression by monocytes in HC (*n* = 5) and RA patients (*n* = 14). Expression was normalized to β-actin, and the comparative Ct method was used for the quantification of gene expression. **(C)** Quantitative PCR analysis of SLC7A5 gene expression (*n* = 5) and **(D)** representative immunoblot analysis for SLC7A5 in LPS (100 ng/ml)-stimulated monocytes (*n* = 5). **(E)** Immunoblot analysis for SLC7A5 in monocytes and monocyte-derived macrophages (MDMs; hereafter macrophages) following LPS stimulation for 18 h. **(F)** Quantitative PCR analysis of SLC7A5 gene expression in LPS-stimulated MDMs (*n* = 8). **(G)** Time-dependent gene expression of SLC7A5 in M1 and M2 macrophages which were polarized with LPS + IFN-γ and IL-4 + IL-13, respectively. **(H)** Immunoblot analysis for SLC7A5 expression in M1 macrophages polarized with LPS + IFN-γ for the indicated times (*n* = 3). Scatter plots show the mean ± SEM. *p < 0.01 and **p < 0.01 by two tailed paired *t*-test **(B,C,F)**. ***p < 0.001: compared both SLC7A5-M2 and SLC7A8-M1 at 24 h poststimulation by two tailed paired *t*-test **(G)**.

It has been demonstrated that peripheral monocytes are activated in patients with RA, and thus, their unique regulation of nutrient uptake is likely also involved in order to meet metabolic demands (30–32). Our pilot microarray assay showed that among a majority of AA transporters, SLC7A5 expression was most greatly upregulated by peripheral monocytes from RA patients when compared with those of HCs (Figure 1A). To confirm our microarray data in a secondary cohort, peripheral CD14^+^ monocytes were purified from HCs and RA patients and the level of SLC7A5 mRNA was compared by quantitative PCR analysis. Peripheral monocytes derived from RA patients, who have higher levels of C-reactive protein (CRP: >0.5 mg/dl), had significantly higher expression (5.85-fold) of SLC7A5 mRNA compared with HCs (Figure 1B; *p* < 0.05). In contrast, the expression level of SLC7A8, another transporter for LNAAs, was markedly decreased in monocytes from RA patients (Figure S3 in Supplementary Material). Given its activation-mediated induction by T cells (27), we first tested whether SLC7A5 is also induced by monocytes upon stimulation, such as LPS treatment. As shown in Figure 1C, LPS-activated monocytes exhibited significantly enhanced mRNA expression of SLC7A5 (23.7-fold, *p* < 0.001). Immunoblotting analysis confirmed that SLC7A5 protein expression was markedly elevated at 24 h by monocytes in response to LPS stimulation (Figure 1D). Moreover, LPS-mediated induction of SLC7A5 was observed in macrophages as well as monocytes (Figures 1E,F). Early study showed that SLC family transporter was upregulated by bacterial infection, and its deglycosylation was important for bacterial pathogenesis (33). Consistent with previous finding (34), SLC7A5 is not a glycosylated protein even though N-glycosylation sites have been predicted in its extracellular loop. The SLC7A5-specific band did not change its position (around 40 kDa) by *in vitro* PNGase F treatment in LPS-stimulated monocytes (Figure S1 in Supplementary Material). Since classical pro-inflammatory M1 macrophages differentiate in response to microbial stimuli, such as LPS and IFN-γ (35, 36), we next analyzed SLC7A5 expression under M1/M2 polarizing conditions. The induction of typical M1 and M1-related genes were confirmed by qPCR in M1 and M2 macrophages, respectively (Figure S2 in Supplementary Material). There was striking upregulation of SLC7A5 expression on M1-polarized macrophages (29.01-fold at 24 h poststimulation; *p* < 0.001), whereas SLC7A8 expression was comparable during M1 and M2 differentiation (Figures 1G,H). LPS-mediated SLC7A5 expression was induced in a concentration-dependent manner and its expression in LPS-activated monocytes and macrophages was dramatically suppressed by treatment with the NF-κB inhibitor, QNZ, but not by the ERK inhibitor, TPl2, or the JNK inhibitor, JNK inhibitor II (Figure S4 in Supplementary Material). These results show that upregulation of SLC7A5 expression is dependent on the activation of human monocytes/macrophages.

### SLC7A5-Mediated AA Influx Has an Influence on Cytokine Production in Monocytes and Macrophages

**Figure 2:**
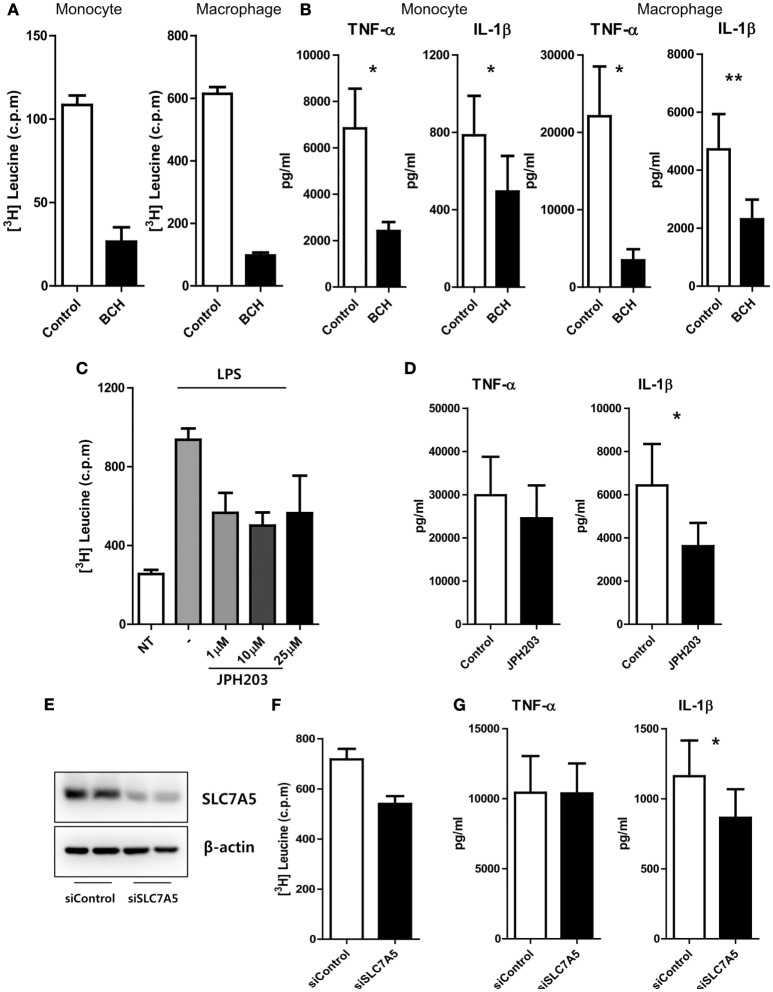
SLC7A5-mediated amino acid (AA) influx has an influence on cytokine production in monocytes and macrophages. **(A)** Uptake of ^3^H-leucine by LPS (100 ng/ml)-stimulated monocytes (left) and macrophages (right) in the presence of 50 mM of BCH, an inhibitor for LAT1 and LAT2. Data are representative of three independent experiments with three different donors. **(B)** The amount of cytokines in culture supernatant from monocytes (left; *n* = 6) and macrophages (right; *n* = 7) upon LPS stimulation. Both cells were stimulated by LPS for 24 h in the absence or presence of 50 mM of BCH. Only macrophages were given additional stimulation with ATP for the last 6 h. **(C)** Uptake of ^3^H-leucine by LPS-stimulated macrophages in the presence of the indicated concentrations of JPH203, a specific inhibitor of LAT1. Data are representative of three independent experiments with three independent donors. **(D)** The amount of cytokines in culture supernatants from macrophages with 10 µM of JPH203 (*n* = 8). **(E)** Knockdown efficiency of SLC7A5. Macrophages were transfected with human SLC7A5-specific or control siRNA (6.6 nM of both siRNAs), and the expression of SLC7A5 was analyzed by immunoblotting 24 h after transfection in the presence of LPS. **(F)** Uptake of ^3^H-leucine by LPS-stimulated macrophages transfected with SLC7A5-specific or control siRNA. Data are representative of three independent experiments with three different donors. **(G)** The amount of cytokines in culture supernatant from macrophages transfected with SLC7A5-specific or control siRNA (*n* = 10). At 4 h post-transfection, macrophages were incubated in HBSS for 1 h to deplete AAs and stimulated with LPS for 18 h with additional stimulation with ATP for last 6 h. Bar graphs show the mean ± SEM. *p < 0.05 and **p < 0.01 by two tailed paired *t*-test **(A–D,F,G)**.

We next sought to investigate modulatory roles of SLC7A5-mediated AA influx on the immune responses of monocytes and macrophages. An AA uptake assay clearly showed that LPS-activated monocytes and macrophages efficiently incorporated ^3^H-labeled leucine and methionine (Figure 2A; Figure S5 in Supplementary Material), and as expected, this uptake of ^3^H-leucine was greatly reduced in the presence of BCH, a general inhibitor of LAT1 and LAT2 (Figure 2A). Of note, while LPS stimulation induced a marked increase in monocyte and macrophage production of pro-inflammatory cytokines, such as TNF-α and IL-1β (Figure 2B), inhibition of SLC7A5-mediated AA influx by BCH (2-amino-2-norbornanecarboxylic acid) led to a marked reduction of TNF-α and IL-1β production by monocytes (2,415 ± 385 pg/ml for TNF-α and 495 ± 183.5 pg/ml for IL-1β) and macrophages (3,467 ± 1,444 pg/ml for TNF-α and 2,307 ± 678 pg/ml for IL-1β) when compared with the untreated group (6,840 ± 1,713 pg/ml for TNF-α and 785.4 ± 203.6 pg/ml for IL-1β in monocytes; 22,099 ± 6,427 pg/ml for TNF-α and 4,715 ± 1,214 pg/ml for IL-1β in macrophages). Although SLC7A8 expression was minimal in LPS-treated monocytes and macrophages, we could not rule out the effect of SLC7A8-mediated AA influx in this experimental setting. Therefore, we next used JPH203, a novel inhibitor specific for LAT1. Consistent with the findings in Figures 2A,B, treatment of macrophages with JPH203 blocked the influx of ^3^H-leucine, resulting in diminished production of IL-1β, but not of TNF-α, in monocytes and macrophages (Figures 2C,D). We corroborated this finding in an SLC7A5 siRNA (siSLC7A5) knockdown experiment (Figures 2E–G). Human monocyte-derived macrophages (HMDMs) were transfected with SLC7A5-targeted or scrambled siRNA and then stimulated with LPS for 18 h. As seen by immunoblotting, the macrophage LAT1 was efficiently silenced (approximately 75% reduced), and this led to a reduction in leucine uptake (Figures 2E,F). As a result, knockdown of SLC7A5 had functional consequences on IL-1β (Mean ± SEM: 864.4 ± 203.8 vs 1,161 ± 255.0) but not on TNF-α production in macrophages (Figure 2G). Thus, the production of IL-1β exerts a higher demand for AAs than does TNF-α in human macrophages. Collectively, these data revealed that regulation of the intracellular AA supply through SLC7A5 induction is critical for production of the inflammatory cytokine IL-1β by human monocytes and macrophages.

### Uptake of Leucine Is Essential for mTORC1-Mediated IL-1β Production

**Figure 3:**
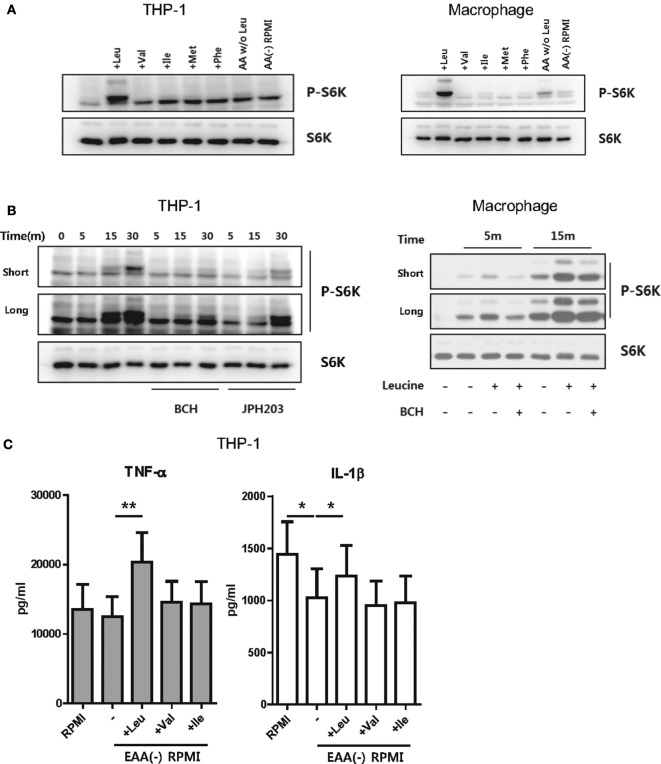
Uptake of leucine is important for mTORC1 activation. **(A)** Immunoblot analysis of phosphor-p70-S6K and total p70-S6K in THP-1 cell lines (left) and human macrophages (right), which were replenished with the indicated essential amino acids (EAAs) (Leu: 50 µg/ml, Val: 20 µg/ml, Ile: 50 µg/ml, Met: 15 µg/ml, Phe: 15 µg/ml). AA W/O Leu indicates leucine-depleted complete RPMI media and AA (−) RPMI indicates a custom-made media depleted of five EAAs (leucine, valine, isoleucine, phenylalanine, and methionine). LPS (100 ng/ml)-stimulated macrophages and PMA-primed THP-1-derived macrophages were incubated with HBSS to deprive amino acids (AAs). Media were changed with a custom-made media depleted of five EAAs. The cells were replenished with the indicated AAs for 15 min and harvested for immunoblot analysis. **(B)** Immunoblot analysis of phosphor-p70-S6K and total p70-S6K in THP-1 cells and macrophages which were replenished with leucine in the presence of 50 mM BCH or 1 µM JPH203 and 10 mM BCH, respectively. **(C)** The amount of cytokines in culture supernatant from THP-1-derived macrophages replenished with the indicated AAs. Cells were stimulated with LPS for 6 h under the indicated culture conditions and additional ATP stimulation for the last 4 h. Bar graphs show the mean ± SEM. *p < 0.05 and **p < 0.01 by two tailed paired *t*-test **(C)**.

Metabolic changes in stimulated monocytes or macrophages are closely linked to production of IL-1β, a critical cytokine in the innate immune response, *via* enhanced glycolysis (10, 37, 38). It also has been reported that mTORC1-S6K activation is responsible for aerobic glycolysis in monocytes and macrophages (39–41). To further investigate how SLC7A5-mediated AA influx influences cytokine production and to determine which AA is required (Figure 2), human macrophages were cultured in media depleted of or replenished with EAA, including leucine, isoleucine, valine, phenylalanine, and methionine, which are transported through SLC7A5. LPS-activated HMDM and THP-1 derived macrophages were incubated in HBSS for 1 and 4 h, respectively, to deprive intracellular AAs and resultantly, mTORC1 became inactivated as embodied by loss of phosphorylation of p70 S6 kinase. As seen in Figure 3A, phosphorylation of p70 S6 kinase was dramatically increased after replenishing leucine. Although LPS-activated THP-1-derived macrophages treated with media, which was replenished with all AAs except leucine, exhibited slightly increased phosphorylation of p70 S6 kinase, this was markedly lower than that seen in leucine-replenished media. Moreover, the leucine-mediated phosphorylation of p70 S6 kinase was markedly blocked by JPH203 as well as BCH (Figure 3B), indicating that mTORC1 activation is induced by LAT-1-mediated leucine influx. We next sought to examine how mTORC1 activation by LAT-1-mediated leucine influx affects cytokine production. IL-1β levels were decreased in culture supernatants from EAA-depleted cultures when compared with those under normal media conditions (Figure 3C). Supplementation with leucine significantly restored the level of IL-1β, as expected with mTORC1 activation, and augmented the level of TNF-α. These results demonstrated that leucine influx through SLC7A5 is linked to cytokine production downstream of mTORC1 activation.

### Inhibition of BCAT-Mediated Leucine Catabolism Enhances Production of IL-1β

**Figure 4:**
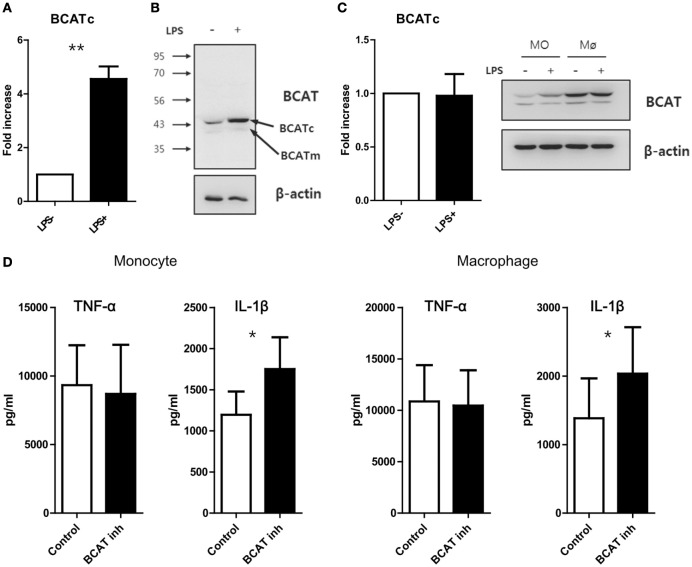
Blockade of catabolism of branched amino acid by BCAT inhibition induces enhanced production of IL-1β. **(A)** Quantitative PCR analysis for BCATc and **(B)** immunoblot analysis for BCAT in monocytes stimulated with or without LPS (100 ng/ml) (*n* = 4). **(C)** Quantitative PCR analysis for BCATc (left; *n* = 5) and immunoblot analysis for BCAT in LPS-stimulated monocytes and macrophages derived from the same donor (right). **(D)** The amount of cytokines in culture supernatants from monocytes (left; *n* = 6) and macrophages (right; *n* = 6). Cells were stimulated with LPS for 24 h in the absence or presence of BCAT inhibitor II. Only macrophages were given additional stimulation with ATP for the last 6 h. Bar graphs show the mean ± SEM. *p < 0.05 and **p < 0.01 by two tailed paired *t*-test **(A,C,D)**.

A recent study demonstrated that the cytosolic branched-chain aminotransferase (BCATc) is an enzyme that regulates the supply of leucine for the mTORC1 pathway in CD4^+^ T cells. Furthermore, the loss of BCATc expression results in increased intracellular leucine concentrations and consequently, enhanced mTORC1 signaling activity and glycolysis in T cells (42). Given the regulatory role of BCATc on leucine concentration, we first measured on BCATc expression in monocytes and macrophages and found that the mRNA level of cytosolic BCAT was strikingly increased in monocytes (Mean ± SEM, 4.55 ± 0.47-fold, *p* < 0.01), but not macrophages, in response to LPS stimulation (Figures 4A,C). In fact, resting HMDMs exhibited higher expression of BCATc protein when compared with LPS-activated monocytes and LPS stimulation of macrophages did not induce any further increase of BCATc expression (Figures 4B,C). In contrast, the expression of mitochondrial BCAT protein, another isoform of BCAT, was unchanged in monocytes and macrophages, irrespective of LPS stimulation (Figure 4C). Considering that BCAT selectively degrades branched amino acids (BCAAs), such as Leu, Val, and Ile (42), and all BCAAs are EAAs which pass through SLC7A5, we sought to investigate whether changes in the intracellular leucine supply *via* inhibition of BCATc activity could modulate mTORC1 signaling activity in human monocytes/macrophages. As seen in Figure 4D, inhibition of enzyme activity using a BCAT-specific inhibitor (BCATc inhibitor 2:5-chloro-2-benzofurancarboxylic acid 2-[[2-(trifluoromethyl)phenyl]sulfonyl]hydrazide) led to significantly increased IL-1β production in LPS-activated monocytes and macrophages, supporting that idea that increased cellular leucine is linked to the enhanced IL-1β production mediated by mTORC1 in human monocytes and macrophages.

### Inhibition of SLC-7A5-Mediated AA Influx Is Associated with Downregulation of Glycolytic Metabolism

**Figure 5:**
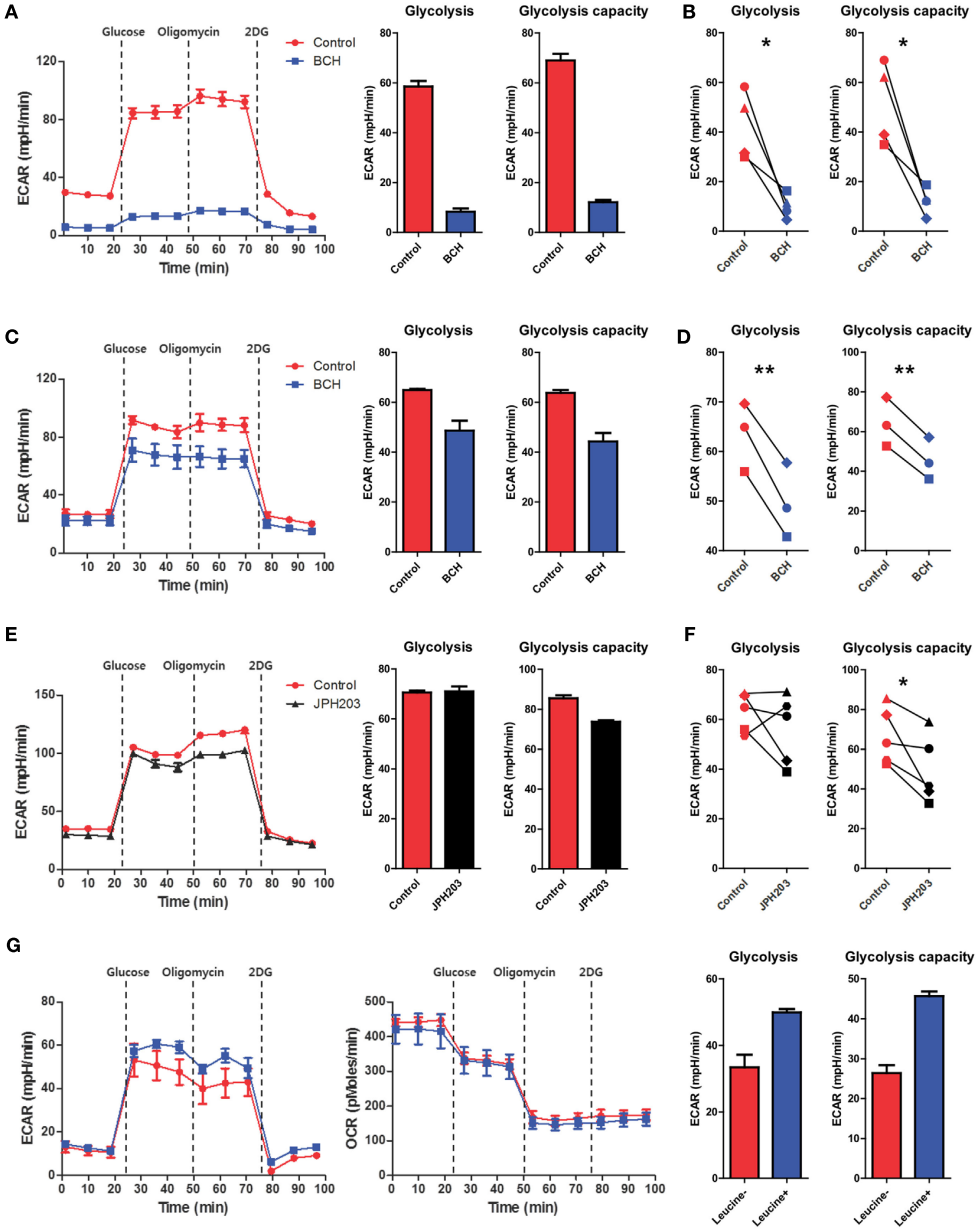
Enhanced glycolysis in activated monocytes and macrophages is related to SLC7A5-mediated amino acid (AA) influx. **(A)** Real-time extracellular acidification rate (ECAR), glycolysis, and glycolysis capacity in monocytes stimulated with 100 ng/ml LPS for 24 h in the absence or present of BCH (50 mM). ECAR levels were measured following sequential treatments with glucose (10 mM), oligomycin (2 µM), and 2-DG (50 mM). Glycolysis and glycolysis capacity were calculated by subtracting ECAR after glucose treatment from ECAR before oligomycin and subtracting ECAR after oligomycin from ECAR before 2-DG treatment, respectively. **(B)** Cellular glycolysis and glycolysis capacity in LPS-stimulated monocytes with or without BCH (50 mM). Monocytes from four different donors were independently tested in at least four technical replicates. **(C–F)** Real-time ECAR, glycolysis, and glycolysis capacity in macrophages stimulated with 100 ng/ml LPS for 24 h in the absence or presence of BCH (50 mM) or JPH203 (10 µM). ECAR levels were measured as described in **(A)**. Monocyte-derived macrophages from three to five different donors were independently tested in at least four technical replicates. **(G)** Real-time ECAR and OCR measurement in LPS-stimulated macrophages replenished with 50 µg/ml leucine in XF assay media. Macrophages were stimulated with LPS for 24 h and were incubated in HBSS for 1 h to deplete AAs. ECAR and OCR levels were measured as described in **(A)**. Data are representative of three independent experiments with three different donors. Bar graphs show the mean ± SEM. *p < 0.05 and **p < 0.01 by two tailed paired *t*-test.

The highly energetic process occurring in LPS-activated monocytes/macrophages induces a shift to glycolytic metabolism to support effector functions (37, 43, 44). To examine whether augmented glycolysis is directly linked to cytokine production by human macrophages, 2-deoxy-d-glucose (2-DG), a general inhibitor for glycolysis, was added to macrophages simultaneously with LPS. Consistent with murine bone-marrow-derived macrophages (BMDMs) and tumor cells (10, 17), inhibition of glycolysis in LPS-activated macrophages significantly reduced the production of IL-1β. Inconsistent with findings in murine BMDMs (10, 17), TNF-α production was also moderately diminished in LPS-activated human macrophage in the presence of 2-DG (Figure S6 in Supplementary Material). Considering the pivotal role of glycolysis in cytokine production, we next asked whether AA influx-mediated mTORC1 activation could modulate glycolytic metabolism. To this end, the ECAR, a parameter for glycolytic metabolism, was measured in monocytes or macrophages activated by LPS in the presence of BCH or JPH203. The ECAR in macrophage and monocytes was markedly decreased by BCH or JPH203 treatment when compared to the untreated control (Figures 5A–F). To observe the specific effect of leucine influx on glycolysis in our experimental setting, LPS-stimulated macrophages were incubated in HBSS for 1 h to deplete AAs, as previously described (27), and replenished with leucine at the beginning of ECAR measurement using XF assays (Figure 5G). Glycolysis and glycolytic capacity were increased only by leucine replenishment with no change in the OCR profile. These data demonstrate that the enhanced glycolysis in LPS-activated monocytes and macrophages might be dependent on SLC7A5-mediated AA influx and especially leucine influx.

### Clinical Relevance of Enhanced SLC7A5 Expression on Monocytes in RA Patients

**Figure 6:**
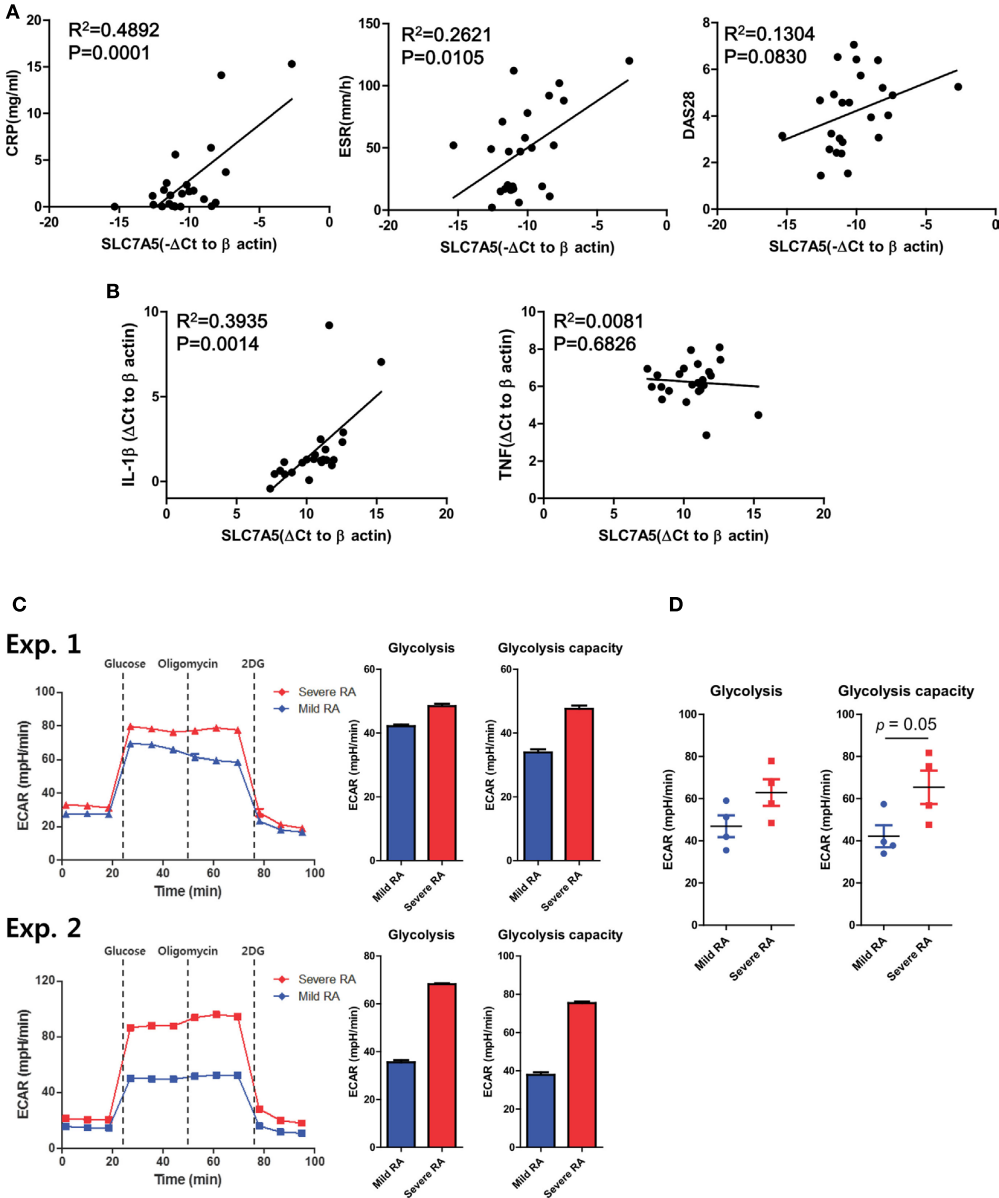
Clinical relevance of increased SLC7A5 expression on monocytes in rheumatoid arthritis (RA) patients. **(A)** Correlation between SLC7A5 gene expression and RA clinical parameters in peripheral monocytes (*n* = 24). Expression was normalized to β-actin and ΔCt was calculated by subtracting the Ct of β-actin from the Ct of SLC7A. The relative gene expression of SLC7A5 is plotted against ESR, CRP, and DAS28. **(B)** Correlation of SLC7A5 gene expression in peripheral monocytes with IL-1β or TNF-α gene expression in RA patients (*n* = 23). *p* Values were obtained using the Pearson correlation analysis **(A,B)**. **(C)** Real-time extracellular acidification rate (ECAR), glycolysis, and glycolysis capacity in monocytes derived from clinically mild or severe RA patients. ECAR levels were measured following sequential treatments with glucose (10 mM), oligomycin (2 µM), and 2-DG (50 mM). **(D)** Scatter plots show glycolysis and glycolysis capacity in peripheral monocytes of mild (*n* = 4) or severe RA patients (*n* = 4). *p < 0.05 and ***p < 0.001 by two tailed unpaired *t*-test **(C,D)**.

Our data thus far suggest that the activation-induced expression of SLC7A5 by monocytes and macrophage contributes to increased production of pro-inflammatory cytokines *via* leucine-mediated mTORC1 activation. Given that SLC7A5 was identified as a candidate gene in monocytes of RA patients, we sought to examine whether elevated expression of SLC7A5 is associated with clinical parameters and disease activity of RA patients. Expression of SLC7A5 by monocytes of RA patients had a significant positive correlation with the serum level of CRP and the ESR, which represent enhanced inflammatory responses (Figure 6A, *p* < 0.012 and *p* < 0.0001, respectively). DAS28 (disease activity score in 28 joints), which represents the disease activity of RA, correlated positively with SLC7A5 expression (Figure 6A, *p* = 0.083). Moreover, there was a significant correlation between SLC7A5 expression by peripheral monocytes and the IL-1β mRNA level in RA patients, suggesting that SLC7A5-mediated metabolic reprogramming may contribute to establishment of the inflammatory milieu in RA disease (Figure 6B, *R*^2^ = 0.3935, *p* = 0.0014). To further investigate the relevance between disease severity and the level of glycolysis, we compared the ECAR measurements in monocytes derived from paired patient sets with mild or severe RA disease (Figure 6C: *n* = 4 paired experiments). The average values of each clinical parameter in the two patient groups were as follows: 0.24 (mild) vs 2.54 mg/dl (severe) for CRP, 13(M) vs 68 mm/h (S) for ESR, and 2.89(M) vs 5.94(S) for DAS28. Remarkably, monocytes from RA patients with severe disease activity showed significantly higher glycolytic capability compared with monocytes of RA patients with mild disease activity. Together, these results suggest that the enhanced SLC7A5 expression by monocytes from RA patients plays a role in the regulation of inflammatory responses in RA *via* increased glycolysis-mediated IL-1β production.

## Discussion

Myeloid lineage cells, such as monocytes and macrophages, undergo specific and complex rewiring of cellular metabolism in response to environmental cues (45). Since activated myeloid cell are central players in a variety of clinical conditions, including infections, autoimmunity, and cancers (3), understanding their metabolic rewiring during diseases may provide insight into disease pathogenesis and result in the identification of viable therapeutic approaches (37, 46, 47). Here, we focused on the AA transporter SLC7A5 and explored its role in modulating pro-inflammatory cytokine production, an important effector function in activated monocytes/macrophages. We found that circulating monocytes derived from RA patients had higher expression of SLC7A5 than did cells from HCs. The induction of SLC7A5 was also observed in LPS-stimulated monocytes and macrophages of HCs, suggesting a possible role for metabolic rewiring during innate inflammatory responses. Further, siRNA knockdown or pharmacological blockade of SLC7A5 reduced IL-1β production by inhibiting leucine-mediated activation of mTORC1, the master regulator of cellular metabolism. Repression of SLC7A5-mediated leucine influx led to downregulation of glycolysis with decreased ECAR. Moreover, the expression level of SLC7A5 on circulating monocytes from RA patients positively correlated with clinical parameters including IL-1β level, suggesting that the SLC7A5-mediated AA influx is related to inflammatory responses in RA.

Rheumatoid arthritis is the prototype systemic autoimmune disease which is characterized by chronic inflammatory responses (48, 49). Although autoreactive T or B cells are essential for development and progress of RA, monocytes/macrophages also play critical roles in the pathophysiology of RA through their expression of costimulatory ligands and production of pro-inflammatory cytokines (4). Several reports have shown that sustained hyper-inflammatory activity of glycolytic macrophages is a central parameter of RA (50, 51), implying that metabolic reprogramming occurs in these cells. A novel finding of our study is that the expression of SLC7A5, a major transporter of EAA, is markedly increased in circulating monocytes in RA patients and this positively correlates with clinical parameters, including IL-1β levels in plasma (Figures 1 and 6). Since SLC7A5 expression is markedly enhanced on activated monocytes and macrophages (Figure 1), it is likely that SLC7A5-mediated AA influx might be involved in metabolic reprogramming. Early microarray studies showed that of SLC family members, SLC7A5 expression is the most greatly upregulated in human monocyte-derived M1 macrophages compared with M2 macrophages (35). Consistent with this finding, M1 polarizing conditions, but not M2-polarizing conditions, greatly induced the expression of SLC7A5 at 24 h (Figure 1G). Of note, enhanced SLC7A5 expression was also observed in peripheral monocytes from patients with gout and end-stage renal diseases (data not shown), suggesting that this induction is a general feature of inflammatory monocytes to fulfill high metabolic demands during immune responses.

Given the conventional role of AAs as substrates for protein synthesis in cell growth and proliferation, the role of SLC7A5 has been primarily investigated in highly proliferative cells such as tumors and T cells, which exhibit rapid proliferation rates (27, 34, 52). In fact, SLC7A5 has been considered a potential therapeutic target for cancer treatment in humans (53–55) and its specific inhibitor, JPH203 (KYT-0353) is currently undergoing a Phase I clinical trial in humans as a novel adjuvant treatment for solid tumors (25). Murine SLC7a5-null T cells have also be found to be unable to metabolically reprogram in response to antigen and cannot not undergo clonal expansion or effector differentiation (27). In contrast to T cells, the effector function of monocytes/macrophages is largely dependent on cytokine production or phagocytosis rather than proliferation (56, 57). Therefore, it is reasonable to investigate how SLC7A5-mediated AA influx modulates cytokine production in activated monocytes/macrophages.

Robust glycolysis is a hallmark of metabolic shift in most immune cells undergoing rapid activation. Despite less effective ways to generate ATP, glycolysis is able to rapidly produce ATP and provide biosynthetic intermediates for supporting effector function (11, 43, 58, 59). Several signaling pathways that trigger glycolysis upon stimulation have been reported in innate myeloid cells (17, 60). For example, in LPS-stimulate macrophages a metabolic intermediate [succinate or the glycolytic enzyme pyruvate kinase M2 (PKM2)] controls activity of hypoxia-inducible factor 1α, which promotes the induction of glycolytic enzymes and inflammatory factors such as IL-1β (17, 60). Of interest, metabolic reprogramming of myeloid cells is particularly more relevant for the production of IL-1β (17). In addition, recent studies suggested that mTORC1 plays a critical role in the activation of cellular glycolysis, which involves the increased translation of glycolytic enzymes or their transcriptional regulators (10). Our study clearly shows that SLC7A5-mediated AA influx in activated monocytes/macrophages leads to augmented cytokine production *via* enhanced mTORC1 activity and glycolysis (Figures 2, 3 and 5). Among AAs which are transported *via* SLC7A5, leucine primarily contributes to controlling mTORC1 activity and phosphorylation of p70 S6 kinase as well as the production of IL-1β and TNF-α in human macrophages (Figure 3).

It has been shown that the activation state of mTORC1, a central regulator of immune cell function, is particularly sensitive to the intracellular levels of certain AAs such as leucine and arginine (61), suggesting that specific AA sensors might exist. Kim and colleagues demonstrated that leucyl-tRNA synthetase (LRS) functions as a leucine sensor for mTORC1 by its activity as a GTPase-activating protein (GAP) for RagD (62). On the other hands, Sabatini and colleagues recently reported that Sestrin2 is a leucine sensor for the mTORC1 pathway. Under leucine-depletion conditions, Sestrin2 is physically associated with and inhibits GATOR2. Upon leucine binding, Sestrin2 is released from GATOR2, allowing it to promote mTORC1 activation through the Rag GTPases (19, 63). LRS is a positive regulator of mTORC1 activation that serves a GAP role for RagD GTPase (62), whereas Sestrin 2 negatively regulates mTORC1 activity by controlling GTP hydrolysis of RagA/B through the regulation of GATOR1–GATOR2 pathway (19, 63). Therefore, in the Rag GTPase cycle, LRS and Sestrin 2 operate as “ON” and “OFF” switches, respectively (64). Our data revealed that leucine has a critical role for recovering mTORC1 activity, which was inactivated under AA starvation conditions (Figure 3A). More importantly, the leucine-mediated mTORC1 activation was linked to production of IL-1β *via* enhanced glycolysis (Figures 3C and 5D). Recent findings demonstrated that asymmetric partitioning of mTORC1 activity and glycolysis after the activation of naive CD8^+^ T cells is attributable to asymmetric partitioning of TCR-induced SLC7A5, which is consistent with the critical role of SLC7A5 in promoting T cell effector functions (65).

The idea of a modulatory role of intracellular leucine on mTORC1 activation in human monocytes/macrophage was supported by our BCATc inhibitor study (Figure 4). Hutson and colleagues reported that BCATc-deficient T cells exhibit upregulated mTORC1 signaling and glycolysis due to the shift of leucine usage from catabolism to mTORC1 activation (42), suggesting a link between leucine metabolism and glycolysis in immune cells. In this study, we found that BCATc was markedly upregulated in activated monocytes and monocyte-derived macrophages (Figure 1) (66). BCATc has been shown to function as an immunomodulatory enzyme limiting Leu supply for mTORC1 by converting it into acetyl Co-A (42). Thus, inhibition of BCATc activity leads to an increased intracellular leucine-supply for the mTORC1 pathway and consequentially, higher IL-1β production *via* glycolytic metabolism (Figure 4D). In contrast to previous reports (42) and our findings, Behmoaras and colleagues recently reported that use of a selective inhibitor for BCATc results in reduced oxygen consumption and glycolysis in LPS-treated macrophages. This reduction is associated with decreased IRG1 levels and itaconate synthesis, suggesting involvement of BCAA catabolism through the IRG1/itaconate axis within the TCA cycle in activated macrophages (66). Although the mechanism underlying this discrepancy is not understood, in our study monocytes/macrophages were stimulated for at least 18 h while the Behmoaras group investigated early (about 3 h) LPS activation and metabolic signatures in murine macrophage (66). Therefore, early metabolic reprograming might be mediated by BCAA influx through SLC7A8, which is constitutively expressed by monocytes and macrophages (Figure S3B in Supplementary Material). Unlike its overexpression in murine activated T cells (27), no obvious upregulation of SLC7A5 gene expression was observed in LPS-stimulated murine peritoneal macrophages or BMDMs (data not shown), suggesting that influx of BCAA including leucine is not facilitated by SLC7A5 in murine macrophages upon stimulation.

One interesting observation in our study was the differential effect on TNF-α and IL-1β production under various experimental conditions. Of note, BCH, which blocks SLC7A5 and SLC7A8, inhibited both TNF-α and IL-1β, whereas both JPH203, a specific inhibitor for SLC7A5, and silencing of SLC7A5 using siRNA preferentially repressed IL-1β and not TNF-α (Figures 2B,D,G). As expected, BCH more efficiently suppressed leucine influx than did JPH203 (Figures 2A,C). Given that metabolic reprogramming of myeloid cells is particularly more relevant for the production of IL-1β (17), it is possible that IL-1β production responds more sensitively to intracellular leucine levels through mTORC1 activation. Further, accumulating evidence has demonstrated that mTORC1-mediated posttranslational control by 4E-BP1 is one mechanism responsible for increased TNF-α production in activated macrophages (67). However, little is known regarding how SLC7A5 expression is transcriptionally controlled in innate immune cells. In tumor cell proliferation, HIF-2α was reported to be a critical transcriptional factor which enhances SLC7A5 expression *via* binding to its proximal promoter (68). However, no obvious elevated expression of the HIF-2α gene was observed in human primary monocytes or macrophages upon LPS stimulation (data not shown). Consistent with previous findings that TCR-mediated AP-1 and NF-κB activation are responsible for SLC7A5 expression in T cells (28), our pharmacological inhibitor assay clearly showed that the induction of SLC7A5 was predominantly dependent on NF-κB activation (Figure S4 in Supplementary Material) *via* TLR/IL-1R-mediated signaling. Given that the SLC7A5 expression surged after 12 h of LPS stimulation, demonstrating that it is a relatively late event following activation (Figure 1G), its expression could be further increased by monocytes/macrophages producing cytokines, such as IL-1β *via* the TLR/IL-1R pathway (Figure S7 in Supplementary Material).

Our findings have clinical importance including that the unchecked or prolonged production of IL-1β is implicated in several acute and chronic inflammatory disorders (5, 69). IL-1β-mediated upregulation of SLC7A5 expression without any obvious external stimuli (e.g., TLR triggering) could explain the increase in SLC7A5 seen in monocytes from patients with inflammatory diseases, such as RA and end-stage renal diseases (data not shown). Considering the correlation between SLC7A5 expression and IL-1β levels in the plasma of RA patients (Figure 6), SLC7A5-mediated IL-1β production by monocytes and macrophages could be a positive loop in chronic inflammatory disorders. Thus, SLC7A5 may be a potential therapeutic target for a variety of inflammatory disorders.

## Ethics Statement

Peripheral blood of RA patients and healthy controls (HCs) was drawn after obtaining written, informed consent. The methods were performed in accordance with the approved guidelines (IRB No. 2012-01-024 for Chungnam National University Hospital and IRB No. 1109-055-378, 1306-002-491, and 1403-049-564 for Seoul National University College of Medicine).

## Author Contributions

BY participated in the design of the study, performed all of the experiments, data collection, and analysis, and drafted manuscript. Y-JO collected patient samples and clinical information and performed data analysis. SK and EL conceived of the study, participated in its design, performed data analysis, and drafted the manuscript. W-WL conceived of the study, participated in its design and coordination, performed data analysis and writing of manuscript, and has full access to all the data in this study and financial support. All authors have read and approved the final manuscript.

## Conflict of Interest Statement

The authors declare that the research was conducted in the absence of any commercial or financial relationships that could be construed as a potential conflict of interest.
