# Supplementary material for: Role of SLC7A5 in Metabolic Reprogramming of Human Monocyte/Macrophage Immune Responses
Source: Front Immunol. 2018 Jan 25;9:53. doi: 10.3389/fimmu.2018.00053 (PMC5788887; doi:10.3389/fimmu.2018.00053)
Supplement: Supplementary file 1 [file Data_Sheet_1.docx]

**Role of SLC7A5 in metabolic reprogramming of human monocyte/macrophage immune responses**

**Authors:** Bo Ruem Yoon, Yoon-Jeong Oh, Seong Wook Kang, Eun Bong Lee and Won-Woo Lee

**Supplementary figures**


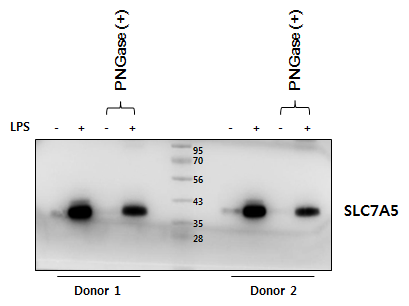


Supplementary Figure 1. SLC7A5 is not a glycosylated protein.

Purified CD14^+^ monocytes derived from healthy donors were stimulated with or without 100 ng/ml LPS for 24 hr. Cell lysate was subjected to i*n vitro* enzymatic deglycosylation with PNGase F (Promega, Madison, WI) for 1 h at 37°C.


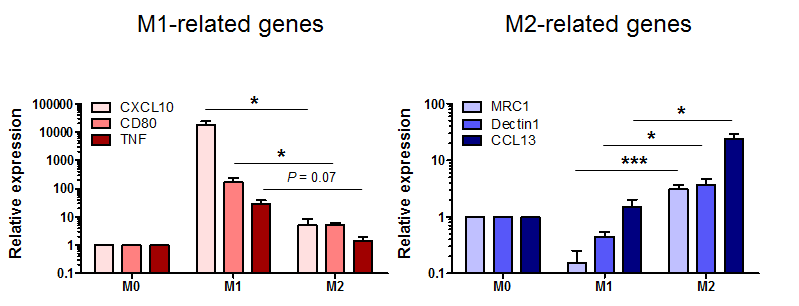


**Supplementary Figure 2. The gene expression profile of M1- and M2-polarized macrophages.**

Human monocyte-derived macrophages (hMDMs) were differentiated from CD14^+^ monocytes in the presence of M-CSF (50 ng/ml) for 6 days. M1 and M2 macrophages were polarized for 24 hr with LPS + IFN-γ and IL-4 + IL-13, respectively. M-1 and M2-related gene expression were evaluated by quantitative PCR analysis (n=4). Expression was normalized to β-actin and the comparative Ct method was used for the quantification of gene expression. Bar graphs show the mean ± SEM. * = *p*<0.05 and *** = *p*<0.005 by paired *t*-test.

**
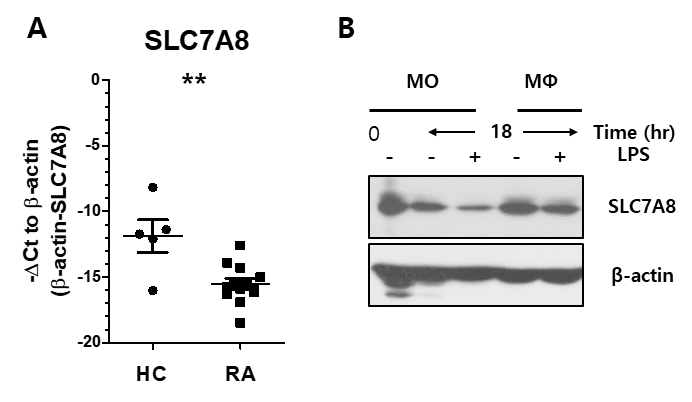
**

**Supplementary Figure 3. The expression of SLC7A8 in human monocytes and macrophages.**

(**A**) Quantitative PCR analysis of SLC7A8 gene expression between HC (n=5) and RA monocytes (n=14), which were the same samples used in Figure 1B. Expression was normalized to β-actin and the comparative Ct method was used for the quantification of gene expression. (**B**) Immunoblot analysis for SLC7A8 in monocytes and monocyte-derived macrophages (MDMs) with LPS-stimulation for 18 hr. The scatter plot shows the mean ± SEM. ** = *p*<0.01 by two-tailed unpaired *t*-test.

**
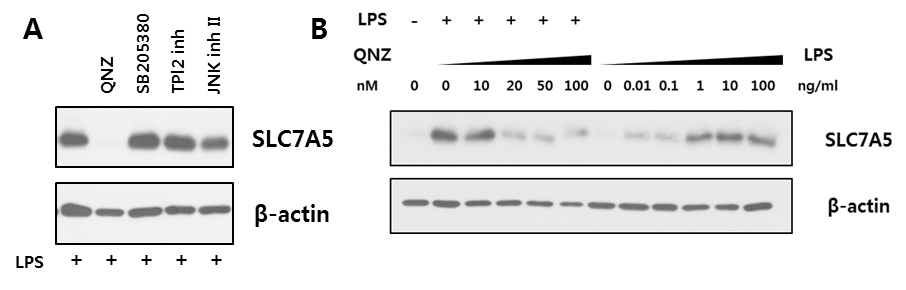
**

**Supplementary Figure 4. Induction of SLC7A5 is dependent on NF-κB activity in human macrophages.**

Immunoblot analysis for SLC7A5 in LPS-stimulated macrophages with the indicated signaling inhibitors. (**A**) Macrophages were cultured for 24 hr with the indicated inhibitors including 50 nM of QNZ, 5 µM of SB205380, 10 µM of Tpl2 inh, or 10µM of JNK inh II(all from Calbiochem, San Diego, CA except SB203580, which was purchased from Cell Signaling Technology) in the presence of LPS. (**B**) Macrophages were culture with the indicated concentrations of QNZ or LPS for 24 hr.

**
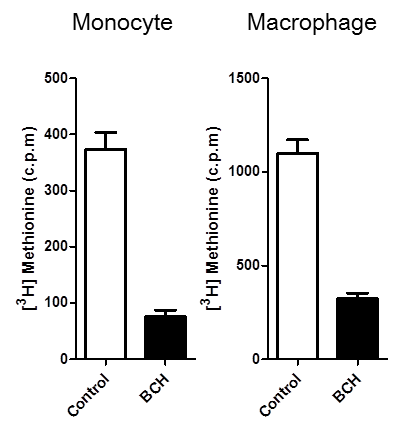
**

**Supplementary Figure 5. Incorporation of methionine by LPS-activated monocytes or macrophages and repression of its uptake by BCH.**

Uptake of ^3^H methionine by LPS-stimulated monocytes and macrophages in the presence of 50 mM of BCH, an inhibitor for LAT1 and LAT2. Data is representative of three independent experiments with three different donors.

**
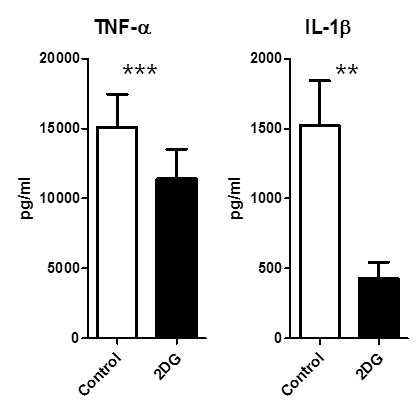
**

**Supplementary Figure 6. Blockade of glycolysis in human macrophages downregulates the production of TNF-α and IL-1β upon LPS-stimulation.**

Cytokine levels in culture supernatants from macrophages (n=7) following LPS-stimulation. Macrophages were stimulated by LPS for 24 hr in the presence of 0.5 mM of 2-DG and given additional stimulation with ATP for the last 6 hr. Bar graphs show the mean ± SEM, ** = *p<*0.01, *** = p<0.001 by two tailed paired *t*-test.

**
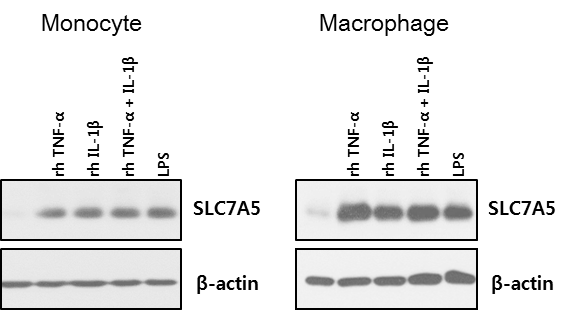
**

**Supplementary Figure 7. SLC7A5 expression is induced by proinflammatory cytokines primarily produced by activated monocytes and macrophages.**

Immunoblot analysis for SLC7A5 in monocytes and macrophages after stimulation with cytokines or LPS. Cells were stimulated with 25 ng/ml of rhTNF-α (R&D systems, Minneapolis, MN) and/or 25 ng/ml of rhIL-1β (Peprotech, Rocky Hill, NJ), or LPS for 24 hr.
